# Mechanism of Acetylcholine Receptor Cluster Formation Induced by DC Electric Field

**DOI:** 10.1371/journal.pone.0026805

**Published:** 2011-10-25

**Authors:** Hailong Luke Zhang, H. Benjamin Peng

**Affiliations:** Division of Life Science and State Key Laboratory of Molecular Neuroscience, The Hong Kong University of Science and Technology, Clear Water Bay, Kowloon, Hong Kong; National Institutes of Health, United States of America

## Abstract

**Background:**

The formation of acetylcholine receptor (AChR) cluster is a key event during the development of the neuromuscular junction. It is induced through the activation of muscle-specific kinase (MuSK) by the heparan-sulfate proteoglycan agrin released from the motor axon. On the other hand, DC electric field, a non-neuronal stimulus, is also highly effective in causing AChRs to cluster along the cathode-facing edge of muscle cells.

**Methodology/Principal Findings:**

To understand its molecular mechanism, quantum dots (QDs) were used to follow the movement of AChRs as they became clustered under the influence of electric field. From analyses of trajectories of AChR movement in the membrane, it was concluded that diffuse receptors underwent Brownian motion until they were immobilized at sites of cluster formation. This supports the diffusion-mediated trapping model in explaining AChR clustering under the influence of this stimulus. Disrupting F-actin cytoskeleton assembly and interfering with rapsyn-AChR interaction suppressed this phenomenon, suggesting that these are integral components of the trapping mechanism induced by the electric field. Consistent with the idea that signaling pathways are activated by this stimulus, the localization of tyrosine-phosphorylated forms of AChR β-subunit and Src was observed at cathodal AChR clusters. Furthermore, disrupting MuSK activity through the expression of a kinase-dead form of this enzyme abolished electric field-induced AChR clustering.

**Conclusions:**

These results suggest that DC electric field as a physical stimulus elicits molecular reactions in muscle cells in the form of cathodal MuSK activation in a ligand-free manner to trigger a signaling pathway that leads to cytoskeletal assembly and AChR clustering.

## Introduction

The formation of the neuromuscular junction (NMJ) has been extensively studied as a model for understanding synapse development. During the assembly of vertebrate NMJ, acetylcholine receptors (AChRs) are clustered to a very high density of ∼10,000 per µm^2^ in the postsynaptic membrane of the skeletal muscle, about 1,000-fold higher than that at extrajunctional membrane [1]. This key event in NMJ development is mediated by the activation of muscle-specific tyrosine kinase receptor MuSK during motor innervation [2]. Recent studies have shown that motoneuron-secreted heparan-sulfate proteoglycan agrin locally activates MuSK via its binding to another transmembrane receptor LRP4 [3]–[4]. The activation of MuSK leads to the assembly of a cytoskeletal scaffold that, together with AChR-associated protein rapsyn, causes AChR clustering [5]–[6]. The diffusion-mediated trapping process that depicts the immobilization and concentration of freely diffusing AChRs in the muscle membrane by a local trapping mechanism induced by the nerve has been widely accepted as explanation to the cluster formation mechanism [7]–[8]. Experimental evidence supporting this model has come from studies using the fluorescence recovery after photobleaching (FRAP) technique in the past [9]–[11] and from quantum dot-aided single AChR tracking more recently [12]–[13].

In addition to motor innervation, AChR clustering can also be induced by a number of non-neuronal stimuli, such as bath or local application of agrin [14]–[16] and growth factor-coated beads [17]–[20]. Direct-current (DC) electric field is another intriguing stimulus in causing AChR aggregation [21]–[24]. When cultured muscle cells are exposed to DC current applied through the medium, they develop AChR clusters at the cathode-facing edge of the cell with high fidelity. The molecular mechanism of this phenomenon is not well understood. It has been explained either by the “electromigration” [24]–[25] or the “diffusion-trapping” [23] model. In the “diffusion-trapping” models, unlike electromigration, there is no directional movement of AChRs and their clustering depends on the immobilization of freely moving AChRs into a cathodal cytoskeletal trap induced by the electric field similar to that resulting from innervation as described above. Previous studies have shown that DC electric field indeed locally activates intracellular signaling pathways as shown by the accumulation of cytoskeletal and tyrosine-phosphorylated proteins along the cathodal edge [23], [26]. Interestingly, DC electric field-induced keratinocyte migration requires the redistribution of activated epidermal growth factor receptors [27], raising the possibility that asymmetric activation of kinases, such as MuSK, underlies the effects of electric field on AChR clustering.

Quantum dots (QDs) are nanometer-sized particles made from semiconductor materials which exhibit high and stable fluorescence without photobleaching, making them ideal probes for tracking single molecules [28]. Using QDs conjugated with α-bungarotoxin (BTX) that binds to the AChR with high specificity, the movement of single receptors at the cell surface under DC electric field was followed. Our findings suggest that the electric field-induced AChR clustering can be explained by “diffusion-trapping” at the cathodal edge. We then further investigated the molecular mechanism underlying this process and found that the action of DC electric field was convergent with that of motor innervation on MuSK activation that led to AChR clustering by a rapsyn and cytoskeleton-dependent mechanism.

## Results

### AChR movement and clustering under the influence of DC electric field

**Figure 1:**
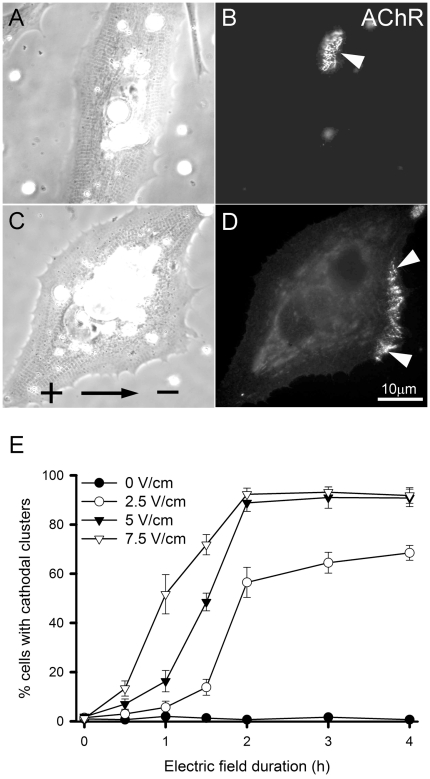
The effect of DC electric field on AChR clustering along the cathodal edge of muscle cells. AChR clusters formed spontaneously in non-innervated muscle cells. The position of these receptor “hotspots” could not be predicted as in control cultures (A, B). DC electric field (direction indicated by arrow in C from anode + to cathode -) caused the formation of AChR clusters along the cathode-facing edge of the cell (arrowheads in D). A and C are phase images and B and D corresponding R-BTX images. (E) The time and electric field-strength-dependence of AChR clustering. Increasing field strength accelerated the clustering process with half-maximum response reached after 2, 1.5 and 1 hr at field strength of 2.5, 5 and 7.5 V/cm, respectively. In control cells not exposed to electric field, no polarized appearance of AChR clusters was seen.

In response to DC electric field, AChRs became clustered along the cathode-facing edge of cultured Xenopus muscle cells as shown by R-BTX labeling (Fig. 1, C and D). These cathodal clusters were quite different from randomly localized AChR hotspots that form spontaneously in these cells (Fig. 1, A and B). The magnitude of this cluster-inducing effect was dependent on the strength and duration of the electric field (Fig. 1E). About 50% of cells exhibited cathodal clusters after 1, 1.5 and 2 hr in response to field strength of 7.5, 5 and 2.5 V/cm respectively. At field strength of 5 V/cm and above, nearly 100% of exposed cells showed AChR clustering along the cathodal side in 2 hrs. These results show the robustness of the muscle cell's response to DC electric field in AChR clustering as previously reported [21], [23].

**Figure 2:**
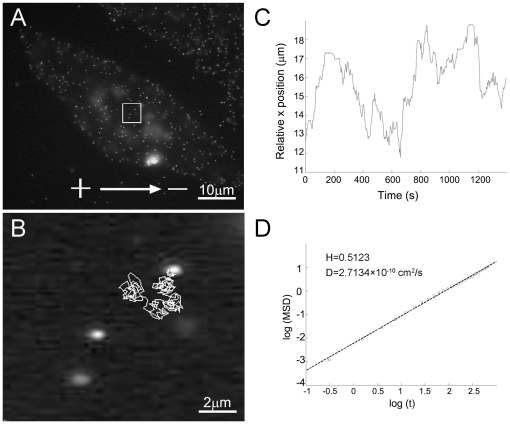
QD-AChR single-particle tracking and trajectory analysis. Single QD-labeled AChRs appeared as bright dots on the surface of muscle cells (A). An example of a 2-D trajectory of AChR single-particle movement under electric field exposure (direction indicated in A) is shown in B, with its projection to the x-axis parallel to the field in C. (D) The plot of MSD and time in logarithmic scale. This plot leads to the calculation of Hurst exponent and diffusion coefficient from the slope and the intercept of the regression line that best-fitted data points as shown. The calculated value of Hurst index is 0.5, indicating that the particle movement conforms to Brownian motion.

**Figure 3:**
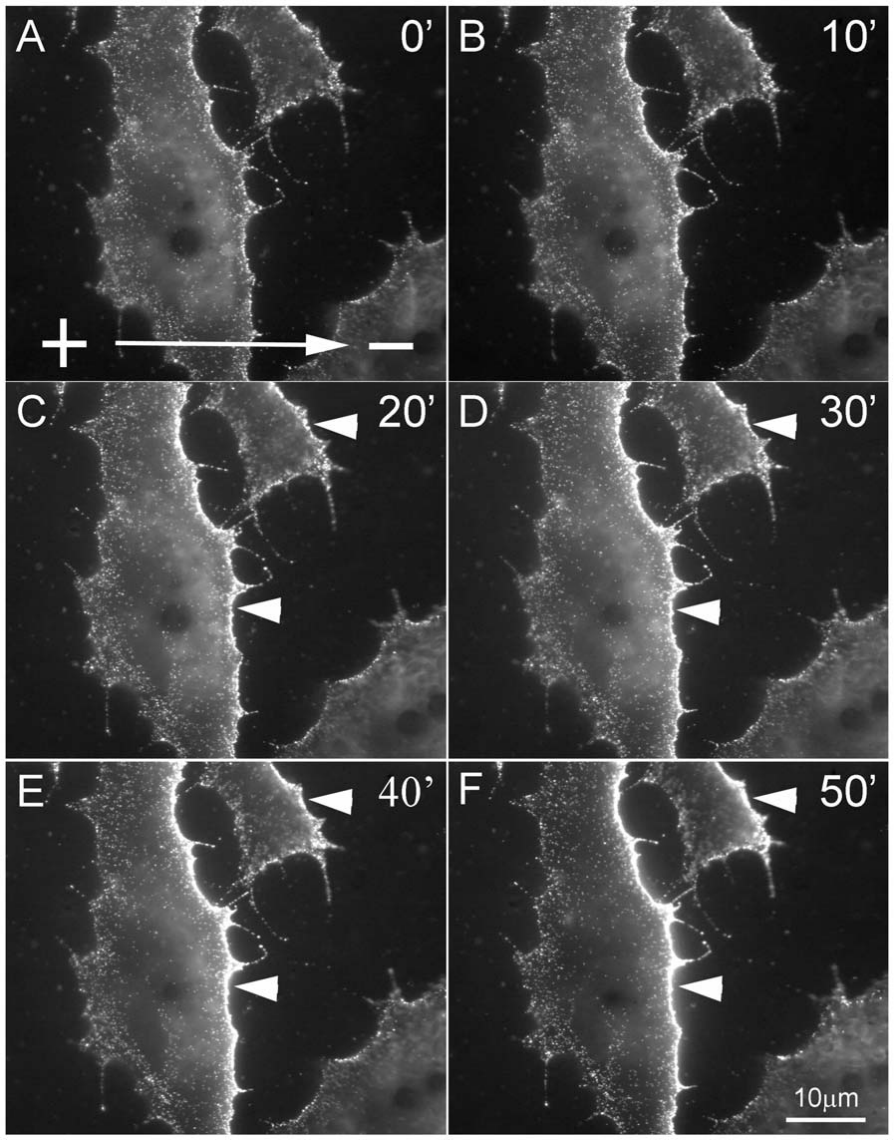
Clustering of QD-labeled AChRs along the cathodal edge of the cell with time. An obvious asymmetry in AChR density as reflected by QD fluorescence intensity was observed after 20 min exposure (arrowheads in C). This asymmetry increased with time.

To analyze the movement of individual AChRs under the electric field, single particle tracking with QDs was employed. AChRs were labeled with streptavidin-conjugated QDs via biotinylated BTX as previously described [12]. As shown in Fig. 2 (A, B) individual AChRs became visible as brightly fluorescent particles undergoing Brownian motion at the cell surface. In response to the electric field, QD-labeled AChRs became visibly clustered along the cathode-facing edge of the cell within 20 min (Fig. 3). Subsequently, the density of QD-AChRs on this edge increased with time, resulting in highly asymmetric distribution with respect to the anode-facing edge of the cell. To analyze single AChR movement, *MSD* was calculated from time-lapse recordings. This allowed us to calculate the diffusion coefficient of AChRs. An example is shown in Fig. 2 (C, D). Here the QD-linked AChR position along the direction of the field (x-axis) was plotted against time (Fig. 2C). From the one-dimensional (x-axis) trajectory, *MSD* values were calculated and from these values, the diffusion coefficient *D* and Hurst exponent *H* (explained below) were also calculated (Fig. 2D).

**Figure 4:**
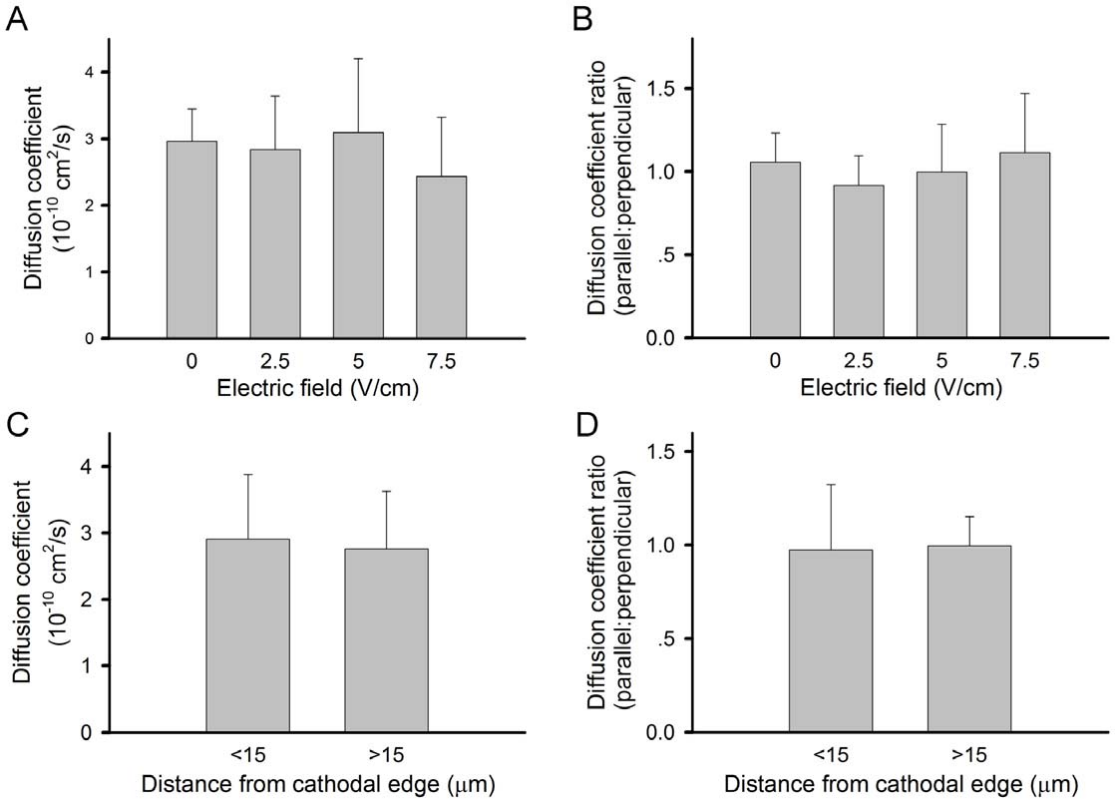
QD-AChR diffusion coefficients. (A) Overall diffusion coefficients. (B) The trajectories were broken down into two components, parallel and perpendicular to the electric field direction and the ratios of respective 1D movement were calculated. (C) Trajectories of QD-AChRs located within or beyond 15 µm of the cathodal edge where receptors were clustered were used in the calculation. (D) Again, trajectories broken down into components parallel and perpendicular to the field direction were used to calculate the ratios. For each bar, more than 50 trajectories were analyzed. Error bars are SEM. No difference in diffusion coefficients under different field strengths, proximity to the cathodal edge or trajectory orientation with respect to the electric field was observed. (*p*>0.1)

As DC electric field is a vectorial stimulus, the AChR movement could be broken down into two perpendicular components, one along the direction of the field (x-axis) and the other perpendicular to it (y-axis). Diffusion coefficients were calculated for movement along either axis. The mean diffusion coefficients for AChRs' movement along the field direction were in the range of 2.5–3x10^−10^ cm^2^/sec and there were no significant differences at all field strengths tested, from 0–7.5 V/cm (Fig. 4A). No difference between parallel and perpendicular movement was detected either (Fig. 4B). We further analyzed the receptor movement near or far away from the cathodal edge and found the diffusion coefficients to be the same irrespective of the receptor's proximity to the cluster-developing region (Fig. 4 C, D).

**Figure 5:**
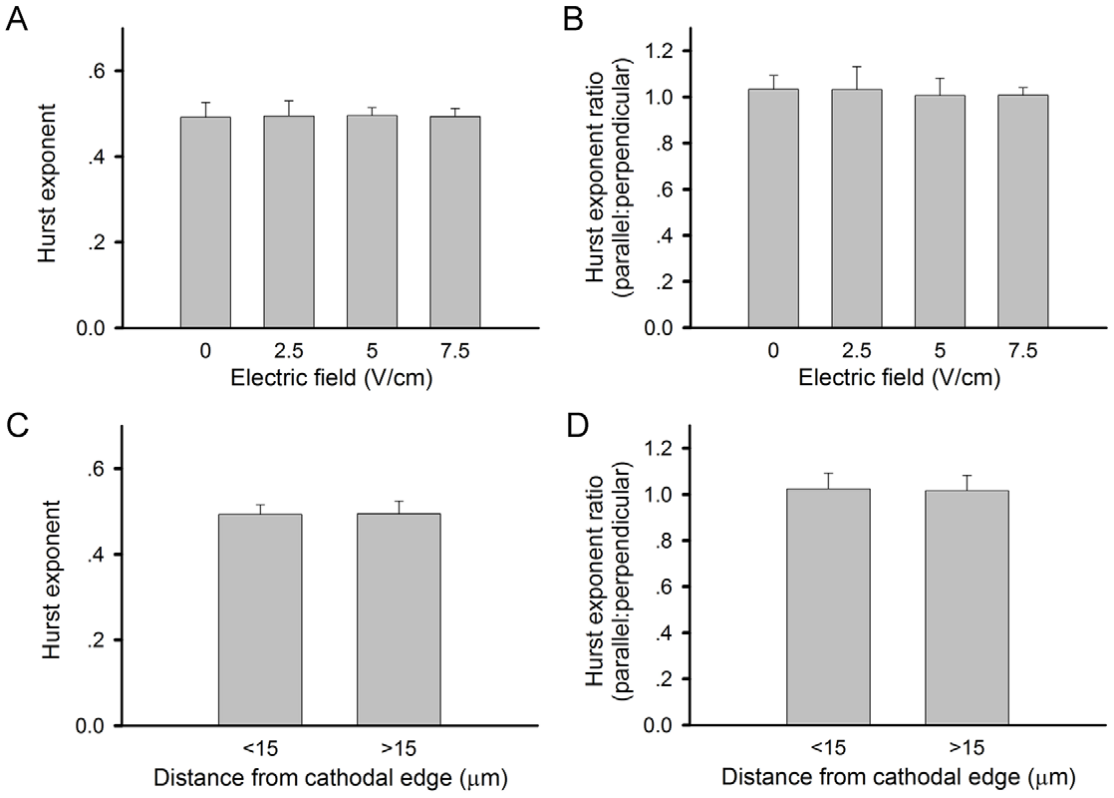
Hurst exponents of QD-AChRs. (A) Overall values calculated from QD-AChR trajectories. (B) Trajectories were broken down into components parallel and perpendicular to the electric field. (C-D) Analyses of trajectories close to or away from cathodal clustering edge. Despite differences in field strength, proximity to the clustering edge and trajectory orientation, the mean Hurst exponent values were uniformly at 0.5, indicating the motion of QD-AChRs is truly Brownian in nature under all conditions. Analyses of each data bar were based on at least 50 trajectories. Indicating lack of differences, the *p*-values are greater than 0.1.

In addition to the diffusion coefficient, Hurst exponent (*H*) was calculated as another parameter to describe the complexity of AChR movement trajectory. This index, with a range of 0.5 to 1, can be used as an indicator of the directional movement of particles under diffusion. When *H = 0.5*, the particle movement is truly Brownian in nature. If *H* is in the range of 0–0.5, the movement is described as “subdiffuion” i.e., *MSD* grows slower than linearly with time and the particle has the tendency of moving “backward” in subsequent steps. On the other hand, if *H* is in the range of 0.5–1, it is called “superdiffusion”, i.e., *MSD* grows faster than linearly with time, as the particle tends to move “forward” in subsequent steps. Similar to diffusion coefficient calculation, AChR movement was also analyzed in parallel and perpendicular to the field direction. As shown in Fig. 5A, the mean Hurst exponent of AChR movement parallel to the field was close to 0.5 at all field strengths ranging from 0–7.5 V/cm. Neither was there any difference between movements parallel and perpendicular to the field (Fig. 5B). We further analyzed the receptor movement near or far away from the cathodal edge and found *H* to be at the value of 0.5 along both axes irrespective of the receptor's proximity to the clustering region (Fig. 5 C and D). These data indicate that AChR movement is described by Brownian motion irrespective of its orientation to the field, the field's strength and its proximity to the cluster-forming region.

### Diffusion-mediated trapping of AChRs under electric field

**Figure 6:**
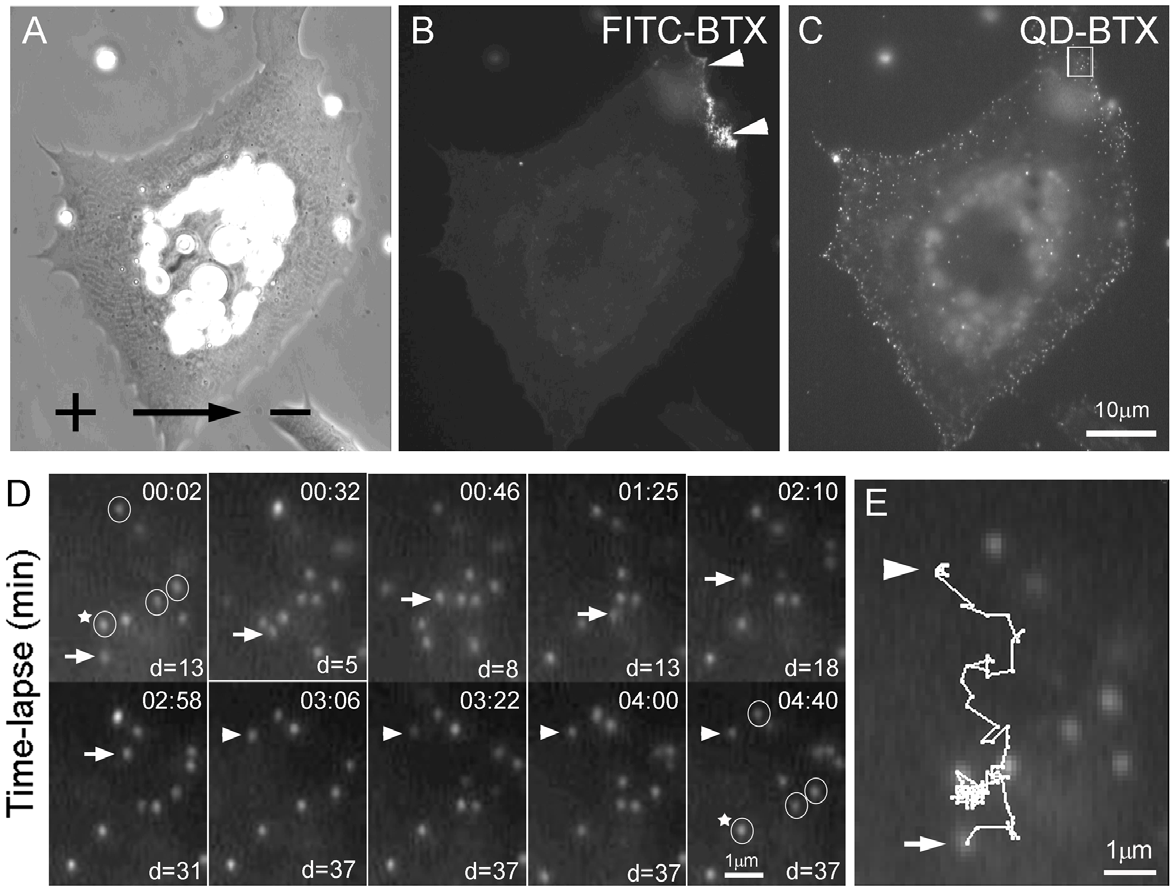
Diffusion-mediated trapping of QD-AChRs on the surface of a muscle cell exposed to DC electric field. FITC-BTX was used to mark the position of the developing cluster (B). QD-labeled AChRs within the developing cluster were followed (region marked by rectangle in C). (D) Time-lapse recording of QD-AChRs within the region. During this approximately 5 min recording, some QD-AChRs were stationary and never moved (encircled). A moving receptor (marked by white arrow) showing Brownian motion during the first 3 min became immobilized and remained stationary after 3 min (indicated by white arrowhead). A stationary AChR (indicated by a star) was used as a reference point from which the distance of the mobile receptor (arrow or arrowhead in each panel) was measured (shown as d values, in pixels). (E) The trajectory of the mobile AChR shown in panel D is shown in higher magnification with the starting and stopping positions shown by arrow and arrowhead, respectively.

Single-molecular tracking with QDs has recently provided direct evidence for diffusion-mediated trapping model for AChR cluster formation in cultured muscle cells induced by motor innervation [12]. To understand the behavior of AChRs as they became clustered along the cathodal edge under DC electric field, time-lapse recording was carried out to track the receptor movement at sites of cluster formation. After exposing cells to 5 V/cm DC electric field for 30 min, developing clusters along the cathodal edge were identified by FITC-BTX labeling and single AChRs labeled with QDs were tracked by time-lapse recording (Fig. 6A-C). As shown in Fig. 6D, some receptors within a cluster-forming area were immobile at the beginning of the observation period (circled QDs) while others underwent Brownian motion within the 5-min recording period (with the arrows pointing to a mobile receptor within this area). It underwent continuous movement for 3 min before suddenly stopping at that time point (Fig. 6D, arrowhead pointing to the immobilized QD that was mobile before the 3 min mark indicated by arrows). It remained immobile for the rest of the recording. Fig. 6E shows the trajectory of this QD-labeled AChR during this period. Recordings like these support the notion that the DC electric field led to cathodal AChR clustering by inducing a trapping mechanism that immobilizes and concentrates freely diffusing AChRs.

### The involvement of rapsyn and actin polymerization in field-induced AChR clustering

**Figure 7:**
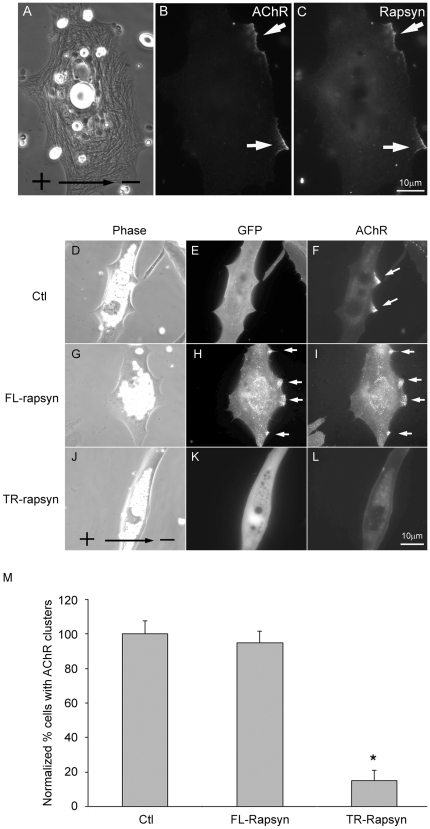
The role of rapsyn in DC-electric field induced AChR clustering. (A-C) Colocalization of rapsyn with electric field-induced AChR cluster along the cathodal edge of the muscle cell. (E-G) GFP expression did not perturb AChR clustering and rapsyn colocalization. (H-J) Exogenous expression of a full-length GFP-tagged rapsyn did not affect AChR clustering. (K-M) Expression of a GFP-tagged rapsyn whose coiled-coil AChR-interacting domain was deleted. This abolished the electric field-induced AChR clustering. (N) Quantification of these results. Number of cells scored: 89 for control cells, 76 for GFP-tagged full length rapsyn and 70 for GFP-tagged truncated rapsyn. Error bars are SEM. * *p*<0.001.

To further understand the molecular mechanism involved in the induction of AChR clustering by DC electric field, we first examined the function of rapsyn. This AChR-associated protein plays an essential role in the cluster formation. Our previous study [26] showed that like nerve-induced AChR clusters, rapsyn is colocalized with cathodal clusters as also shown here in Fig. 7 (A-C). Rapsyn consists of N-terminal myristoylation domain, seven tetratricopeptide-repeats (TPRs) and C-terminal coiled-coil domain and the deletion of the coiled-coil domain alone resulted in failure of AChR clustering when assayed in HEK293T cells [29]. Thus, GFP-tagged full-length or mutant rapsyn with coiled-coil domain deletion was expressed in Xenopus muscle cells to study the function of this protein in electric field-induced AChR clustering. Control cells expressing GFP alone showed normal field-induced cluster formation (Fig. 7 D-F). The exogenous full-length rapsyn did not interfere with field-induced AChR clustering and like the endogenous protein, was also colocalized with the receptors at the cathode (Fig. 7 G-I). Cells expressing truncated rapsyn devoid of coiled-coil domain, however, failed to form AChR clusters along the cathode and the mutant protein did not localize along the cathodal edge (Fig. 7 J-L). These results, quantified in Fig. 7M, suggest that rapsyn is involved in DC field-induced AChR clustering.

**Figure 8:**
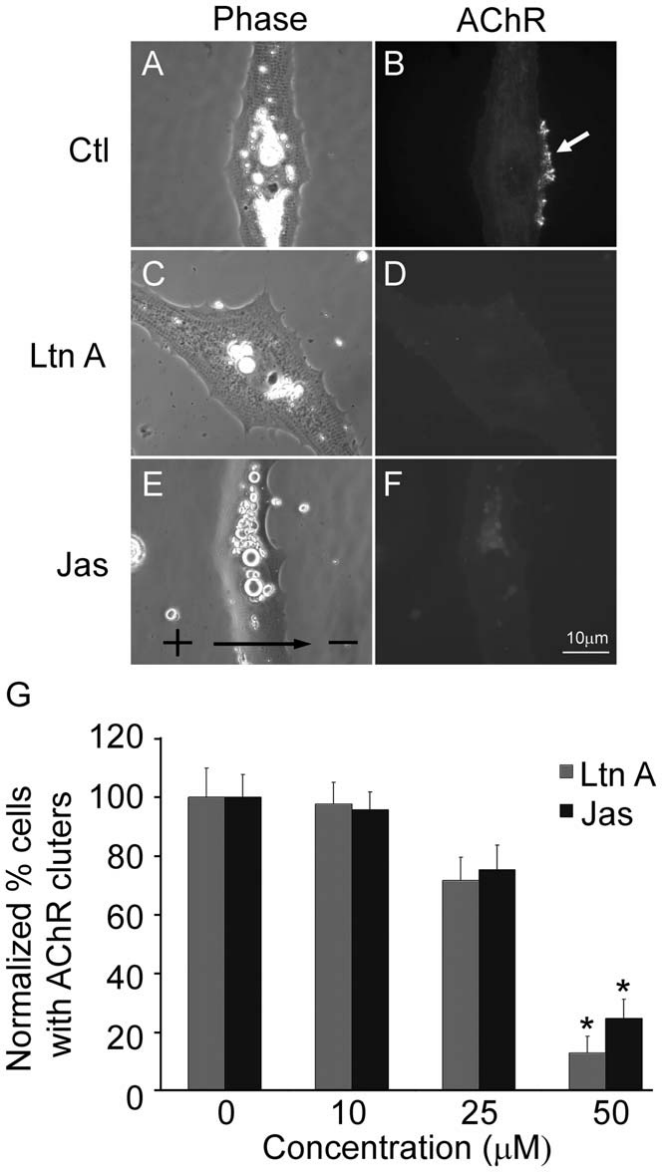
The effect of perturbing actin polymerization on electric field-induced AChR clustering. (A, B) A control cell showing cathodal AChR clustering. (C, D) Latrunculin A (LtnA) at 50 µM abolished electric field-induced cluster formation. (E, F) Jasplalinolide (Jas) at 50 µM also inhibited this process. Cells were pretreated for 1 hr and then maintained in medium containing LtnA or Jas throughout electric field application. (G) Quantification shows dose-dependent inhibition of cluster formation by these actin-disrupting toxins. More than 50 cells were examined in each treatment. * *p*<0.001.

As local F-actin cytoskeleton assembly is necessary for AChR clustering induced by a number of stimuli such as agrin and growth factor-coated beads [30]–[32], we also examined its involvement in electric field-induced cluster formation. LtnA is a marine sponge toxin that binds to G-actin and potently blocks its polymerization into F-actin [33]. We found that cells treated with this toxin failed to form cathodal AChR clusters in response to DC electric field in contrast to the control (Fig. 8 A-D and G). In addition, the effect of jasplakinolide was also tested. This is another marine sponge toxin that causes polymerization and stabilization of F-actin in a manner not controlled by cellular signaling [34]–[35]. It also interfered with cluster formation induced by DC electric field in a dose-dependent manner (Fig. 8 E-F and G). These results support the notion that local F-actin assembly is also involved in AChR clustering induced by DC electric field, suggesting that it activates a local signaling mechanism for cytoskeletal assembly.

### Local kinase activation induced by electric field

**Figure 9:**
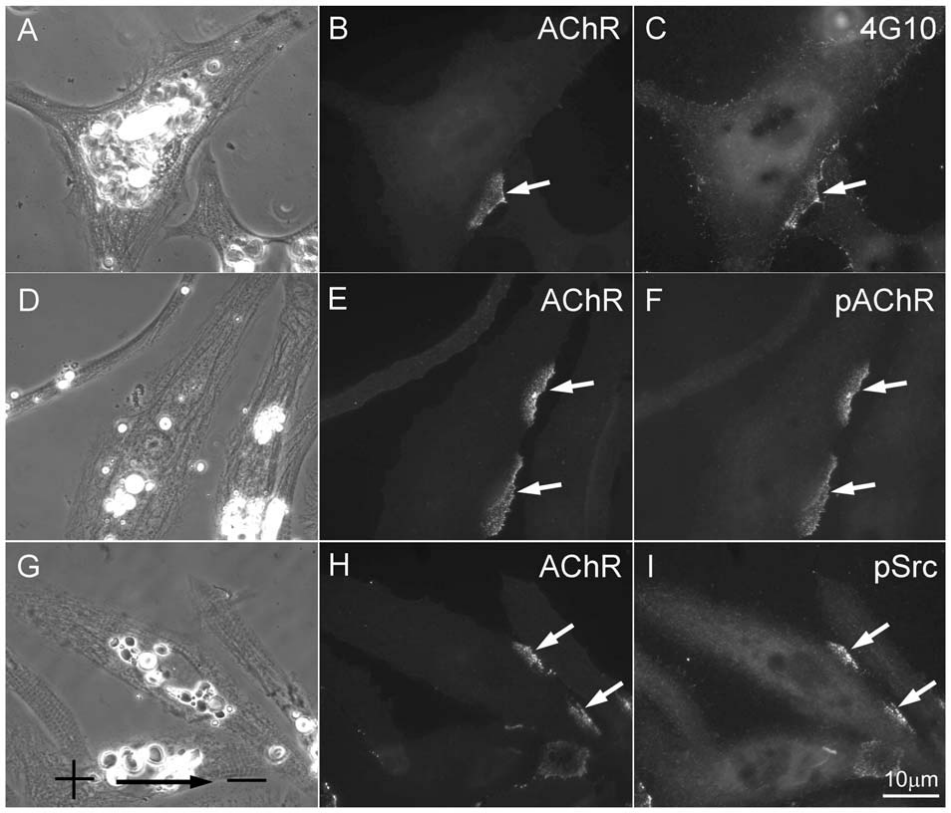
Association of phosphotyrosine proteins at electric field-induced ACh clusters. (A-C) Localization of phosphotyrosine labeling at cathodal AChR clusters. Mab4G10 was used to label phosphotyrosine-containing proteins in C. (D-F) AChR β-subunit phosphorylated on Y390 (F) was prominently localized at cathodal cluster after two hours of exposure to electric field. (G-I) Localization of phospho-Src (pSrc) at cathodal clusters. An antibody that recognizes Src with its Y416 phosphorylated was used to localize active Src. pSrc was localized clearly at cathodal clusters (H and I).

Consistent with our previous finding [23], DC electric field causes tyrosine kinase activation along the cathodal side of the muscle cell as evidenced by labeling with an anti-phosphotyrosine antibody (Fig. 9 A-C). It has been reported that Y390 of AChR β subunit is phosphorylated after synaptogenic stimulation and this is crucial for AChR cluster maintenance [36]. Using anti-phospho-AChR β-subunit antibody specific for Y390 (pAChR), the cathodal localization of this signal was also observed. As shown in Fig. 9 (D-F), phosphor-β-subunit was colocalized with AChRs as shown by fluorescent BTX labeling.

To further understand the upstream kinase activation that leads to AChR β-subunit phosphorylation, we turned our attention to Src kinase as it has been shown to phosphorylate this subunit upon MuSK activation [37]–[40]. Src appeared to distribute evenly in cells with or without DC electric field exposure as seen with a pan-Src antibody (data not shown). However, phosphorylated Src (pSrc), labeled with an antibody that recognized this kinase with its Y418 residue phosphorylated, was highly concentrated along the cathodal edge of the cell and colocalized with AChR clusters (Fig. 9 G-I). Y418 is located within the catalytic domain of Src and its phosphorylation is accompanied by high enzymatic activity of this kinase [41]. Together, these data suggest that DC electric field causes asymmetric tyrosine kinase activation along the cathodal edge of muscle cells. This step is necessary for AChR clustering as shown in our previous study [23] and in the study described below.

### The involvement of MuSK

Results presented above suggest that the electric field-induced AChR clustering, like that initiated by agrin/MuSK stimulation, involves tyrosine kinase activation and cytoskeletal assembly. As MuSK activation is essential for postsynaptic development at the NMJ, we set out to test whether it also mediates the electric field effect.

**Figure 10:**
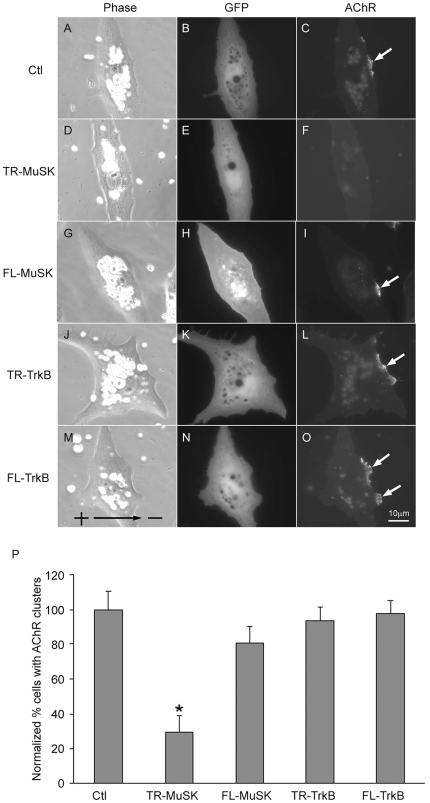
The function of MuSK in electric field-induced AChR clustering. (A-C) Xenopus muscle cells expressing GFP only showed normal cathodal clusters (pointed by arrow on the right edge for the cell). (D-F) The expression of a kinase-dead MuSK abolished AChR clustering. (G-I) Full-length MuSK did not affect the cluster formation on the cathodal side. The expression of Trk B, either with its kinase domain truncated (J-L) or in full length (M-O), did not affect the cell's response to electric field. (P) Quantification based on the following number of cells examined, n = 105 (control), 77 (truncated MuSK), 86 (full-length MuSK), 94 (truncated TrkB) and 72 (full-length TrkB). Error bars are SEM. * *p*<0.01.

The function of MuSK was suppressed through the expression of a truncated version that lacks the tyrosine kinase domain. This construct was expected to produce a dominant-negative effect on MuSK signaling [42]. Muscle cells were prepared from embryos injected with mRNA encoding this mutant MuSK. In this and following studies, GFP mRNA was co-injected for identifying cells expressing the exogenous protein. As shown in Fig. 10 (D-F), muscle cells expressing this kinase-dead MuSK failed to cluster AChRs along the cathodal edge in response to DC electric field as compared to control cells expressing GFP (Fig. 10A-C). Cells expressing full-length MuSK showed normal response to electric field in cathodal AChR clustering (Fig. 10G-I). To further test the specificity of the inhibitory effect of MuSK perturbation, the function of another tyrosine kinase receptor, TrkB, was also manipulated. The expression of either kinase-dead or full-length TrkB had no influence on field-induced AChR clustering along the cathode-facing side (Fig. 10 J-O). These results, quantified in Fig. 10P, showed that truncated MuSK expression alone resulted in a 70% reduction in field-induced receptor clustering. They support the involvement of MuSK activation in electric field-induced AChR clustering and suggest that DC electric field locally activates MuSK along the cathodal edge of the cell in a ligand-independent manner to initiate the signaling cascade for AChR clustering.

## Discussion

Among stimuli that induce the formation of AChR clusters in muscle cells, DC electric field is unique in that its action is not mediated by a direct ligand-receptor interaction. In addition to AChR clustering, this stimulus is known to induce redistribution of ConA and EGF receptors [43]–[44], directional neurite outgrowth [45]–[47] and orientation and migration of a variety of cells types [27], [45], [48]–[53]. The molecular mechanisms underlying these diverse effects are not fully understood, although previous studies have shown DC electric field is capable of cytoskeletal reorganization, cathodal membrane depolarization and calcium influx and local activation of signal transduction [48], [54]–[57]. In this study, we have extended previous studies in elucidating the molecular mechanism of field-induced AChR clustering along the cathodal edge of the muscle cell. First, we found that the electric field induces a “trap” for immobilizing and clustering AChRs along the cathodal edge. Second, this trap is established as a consequence of cathodal MuSK activation and also involves rapsyn localization. These findings suggest a convergence of the signaling mechanism activated by DC electric field and by motor innervation. In the latter case, it has been well established that agrin secreted by motor axons causes MuSK activation to lead to local AChR cluster formation at the nerve-muscle contact.

An alternative to the diffusion-mediated trapping hypothesis in explaining AChR clustering induced by DC electric field is the electromigration model that depicts the redistribution of membrane-bound molecules as a consequence of electrophoresis and electro-osmosis. It was proposed that through electrophoresis, membrane molecules are moved and become sequestered according to their charges by electrical gradient tangential to the cell membrane [58]. On the other hand, electric field can also cause electro-osmotic flow of cell surface fluid that exerts viscous drag of membrane-bound molecules to cause their movement and clustering [25]. To test whether electromigration can also account for electric field-induced AChR clustering, a mathematical simulation of the movement of QD-bound receptors was conducted (Text S1). This simulation integrated the effects of several forces on AChRs, including their collision with membrane lipids, viscous drag from the overlying fluid phase and the externally applied electric field. The results (Fig. S1) showed that the Brownian-type motion of AChRs was unaffected by electric field over the range used in the current study (<10 V/cm). The electromigration effects on cathodal AChR clustering only became obvious when very large field on the order of 10^3^–10^4^ V/cm was applied (Fig. S1). On the other hand, our simulation showed diffusion-mediated trapping model satisfactorily explained cluster formation at low, physiologically relevant field strengths (Fig. S2).

In this study, QDs were used to track single AChRs as they became clustered under the influence of DC electric field. Our results have shown that electric field does not directly act upon the receptors to cause their directional movement and clustering. Receptors both far away and near the cluster-forming site along muscle cell's cathodal edge exhibited Brownian motion under all field strengths tested as shown by Hurst exponent calculation. Their immobilization was visualized as sudden cessation of QD-labeled AChR movement at developing clusters along the cathodal edge. Thus, the diffusion-mediated trapping model can satisfactorily account for electric field-induced AChR clustering. This model was proposed to account for nerve-induced AChR cluster formation more than 30 years ago and has been a widely accepted hypothesis. We recently provided directly evidence based on QD-AChR tracking for nerve and bead-induced clustering processes [12]–[13]. Our current results suggest a common signaling mechanism that can be activated by ligand-mediated stimulus such as motor axon and non-ligand-mediated stimulus such as electric field.

Previous studies have also shown that electric field-induced AChR clustering shares additional features in common with NMJ formation. First, electron microscopy has shown that cathodal clusters are associated basal lamina and cytoplasmic density similar to that found at the NMJ [22]. Similar to NMJ, rapsyn is localized at cathodal clusters [26]. Furthermore, the localization of phosphotyrosine, indicative of tyrosine kinase activation, is a common feature of both nerve and electric field-induced AChR clusters [23]. These striking similarities strongly indicate that a common signaling pathway is stimulated by both motor axon and DC electric field in muscle cells. We have now shown that muscle cells expressing a MuSK mutant devoid of the intracellular kinase domain responded poorly to electric field in cathodal AChR clustering. This suggests that local MuSK activation, which usually takes place along the nerve track during innervation, is also elicited along the cathodal edge and is necessary for electric field to trigger the clustering process. Showing specificity, we found that the role of MuSK can not be replaced by another tyrosine kinase receptor TrkB. In addition, Src kinase activation as shown by phospho-Src antibody labeling, is also localized at electric field-induced clusters. Similarly, agrin secreted by motor axon leads to Src activation [38], [59]–[61]. One of the effectors of Src is the beta-subunit of AChR whose phosphorylation at a key tyrosine residue is required for increasing the stoichiometry of rapsyn binding and the cluster stability [36], [38], [61]–[62]. Using a phospho-AChR-beta-subunit antibody, we indeed observed its localization at cathodal clusters. These results are consistent with previous studies that have also suggested the activation of tyrosine kinase receptors, such as EGF-receptor [54], [63], and intracellular kinases, such as protein kinase C and cAMP-dependent kinase [55], [64], in mediating electric field-induced cellular responses.

The assembly of an F-actin cytoskeleton is also necessary for the electric field effect as shown by the inhibition of cluster formation under LtnA or jasplakinolide treatment. LtnA blocks actin polymerization due to its ability to sequester G-actin [33]. Jasplakinolide, despite its F-actin stabilizing property in vitro, disrupts in vivo actin polymerization by promoting the formation of disordered actin polymers and depleting monomers, thus perturbing cellular functions [34]–[35]. This dependence on F-actin assembly resembles other cellular effects generated by this stimulus. For example, DC electric field induces polarized formation of lamellipodia along the cathodal edge in epithelial cells that are enriched in F-actin and causes their migration toward the cathode [48], [51]. On the effect of AChR-cluster induction, electric field is again similar to other synaptogenic stimuli such as agrin and growth factor-coated beads in their common requirement of local F-actin assembly at developing AChR clusters [30], [32]. This suggests that electric field exerts its effect by causing MuSK activation and actin polymerization along the cathodal edge of the muscle cell. This F-actin cytoskeleton, together with rapsyn, is presumably the basis for immobilizing and clustering AChRs.

In conclusion, this study has provided an understanding on the molecular mechanism in DC electric field-induced AChR cluster formation in muscle cells. Single-molecule tracking of quantum dot-labeled receptors showed that the electric field does not influence their Brownian motion at the cell surface. However, it triggers a local F-actin cytoskeletal specialization along the cathodal edge of the muscle cell that immobilizes and clusters AChRs through rapsyn. The formation of this cathodal “trap” is dependent on MuSK activation. Thus, despite their distinct nature, the motor axon and DC electric field are remarkably similar in their actions on activating MuSK and its downstream intracellular signaling pathway to cause local AChR cluster formation. As discussed above, MuSK is not the only tyrosine kinase receptor that can be activated by electric field. Future studies aimed at uncovering how these membrane-bound receptors can be activated in a ligand-free manner by this physical stimulus will likely reveal novel mechanisms of cellular signaling.

## Materials and Methods

### Cell culture

Primary cultures of Xenopus myotomal muscle cells were prepared as described previously [65]. Briefly, embryos at stage 20–22 were sterilized in 0.02% thimerosal for 5 min and then washed twice with Holtfreter's solution. The jelly coat and vitelline membrane were removed using hypodermic needles and the dorsal parts of embryos were dissected out into Steinberg's solution. After digestion in 1 mg/ml collagenase, myotomes were separated and dissociated in a Ca^2+^/Mg^2+^-free solution and the cells were plated on coverglass coated with entactin-collagen-laminin (ECL) substrate (Upstate Biotechnology-Millipore) in Steinberg's solution, supplemented with 10% L-15 (Leibovitz) medium, 1% fetal bovine serum, 100 U/ml penicillin. The cultures were maintained at 23°C for 1–2 days.

### DC electric field application

A custom chamber was constructed for electric field application to cell cultures by modifying a published design [66]. A 20×20 mm^2^ hole was made on the bottom of a 35 mm Petri dish and covered with a 24×24 mm^2^ #1 coverglass from the inside. Next, two spacer strips were glued to the coverglass to form a 24 mm×5 mm×0.3 mm groove. After placing the tissue culture grown in #1 coverglass to the bottom of the chamber was covered by another piece of coverglass to form a medium-filled space with uniform cross-sectional area. This chamber was connected to Ag-AgCl electrodes via 1% agar-saline bridges and a constant-voltage power supply was used to supply electrical current. The field magnitude was calculated from the voltage drop measured across the chamber. The cells were observed during the electric field application on an inverted microscope.

### AChR labeling, antibody staining and imaging

To examine AChR clusters, muscle cells were stained with tetramethlrhodamine- or FITC-conjugated α-bungarotoxin (R-BTX or FITC-BTX; 3 nM) (Molecular Probes-Invitrogen) for 30 min. To track single AChRs using QDs, previous procedures published by our lab were followed [12]. In short, cells were labeled with biotin-conjugated BTX and Alexa 488-conjugated BTX (both from Molecular Probes-Invitrogen) at concentrations of 0.5 and 25 nM respectively. The culture was then labeled with streptavidin-conjugated quantum dot 655 (QD655 with emission wavelength of 655 nm; from the same company) at the concentration of 2.5 nM. Cells were visualized live on an inverted microscope using a 60x oil-immersion magnification. For some experiments R-BTX-labeled cells were fixed with cold 95% ethanol or with 2% paraformaldehyde in PBS and permeabilized by 0.5% Triton X-100, and after blocking with PBS containing 5% bovine serum albumin, labeled with primary antibodies and FITC-linked secondary antibodies diluted in the blocking buffer.

All images were captured using an Olympus IX70 inverted microscope fitted with a Hamamatsu ORCA II chilled-CCD camera controlled by MetaMorph software (Molecular Devices). A Sutter Instruments Lambda shutter was employed in time-lapse imaging experiments. Optical filter sets specific for rhodamine, FITC or QD655 were used for examining samples with each fluorescent label.

### Single particle tracking

To study single AChR movement at the cell surface, trajectories of QD-labeled AChRs were recorded by time-lapse recording at 3 sec intervals for 30-min each. QDs were followed with the “Track Point” function under MetaMorph software package to decipher the trajectory of each QD-AChR under study and QDs were identified as single particles rather than as small aggregates based on fluorescence intermittency (“blinking”). Only single QDs exhibit the blinking property and this can be used to rule out multiple-QD-AChR aggregates [12]. Because of this “blinking” property, a small number of points along the single particle trajectory were not visible. However, as we previously showed by simulation [12], analyses based on the visible periods yielded results that were indistinguishable from that based on the entire trajectory. A 2-D trajectory was projected to the x-axis in parallel to the electric field direction and to the y-axis perpendicular to it and corresponding 1-D displacement/time graph was generated (e.g., Fig. 2C). Mean squared displacement (MSD) in one dimension was calculated as follows:
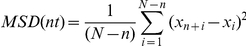
where *x_n+i_,* is the position of the QD-AChR after the time interval of *nt* (*t* is the time interval between successive measurements) following its location at position *x_i_*. *N* is the total number of positions recorded; *n* ranges from 1 to *N-1*.

The Hurst exponent is defined according to Mandelbrot's original fractal theory [67] and Addison's further work [68]:
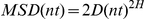
The following equation follows:
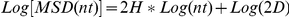
where *D* is fractal diffusion coefficient and *H* is Hurst exponent which is an index describing trajectory complexity (Fig. 2D). *D* and *H* values can be obtained by fitting data with the above equations and analyzed using SPSS software. When *H* equals to 0.5 (i.e., Brownian motion), *D* becomes the diffusion coefficient calculated through *MSD* analysis.

### cDNA constructs and mRNA synthesis and expression

In some experiments rapsyn, MuSK and TrkB proteins were ectopically expressed in muscle cells. Wild-type mouse rapsyn cDNA was kindly provided by Dr. Jean Cartaud (Institut Jacques Monod, CNRS, Paris) and a coiled-coil domain-deletion mutant (Δ297–331) was generated using PCR. Both were linked at C-terminus with green fluorescent protein (GFP) by inserting them into pEGFP-N1 plasmid (Clonetech). Wild type mouse MuSK was a gift of Dr. Alastair Reith (GlaxoSmithKline) and a kinase domain-deleted mutant (1–571) was amplified by PCR, both tagged with myc epitope. Constructs encoding wild-type rat TrkB, kindly provided by Dr. Nancy Ip (The Hong Kong University of Science and Technology), and human TrkB-T1 cDNA without the kinase domain, cloned by Dr. Jie Zhou (The Hong Kong University of Science and Technology), were tagged with HA epitope. All sequences were subcloned into pcDNA3.1(+) vector for mRNA preparation. After linearizing the plasmids, mRNAs encoding these constructs were synthesized using the mMESSAGE mMACHINE kit (Ambion) and injected into 2-cell stage Xenopus embryos using a Drummond Nanojet Oocyte Injector (Drummond Scientific Co.) individually. Injected embryos were allowed to develop to stage 20–22 before cultures of muscle cells were made from them.

### Reagents

Various antibodies were purchased from the following sources: anti-phosphotyrosine monoclonal antibody 4G10 (Upstate Biotechnology-Millipore), rabbit polyclonal antibodies against Y390-phosphorylated AChR β-subunit (Santa Cruz Biotechnology), anti-rapsyn monoclonal antibody and rabbit polyclonal antibody against MuSK (Thermo Fisher Scientific Inc.), phospho-Src (Y416) antibody (Calbiochem) and rhodamine- and FITC-conjugated secondary antibodies (Zymed). Latrunculin A (LtnA) and jasplakinolide (Jas) were from Molecular Probes-Invitrogen.

## Supporting Information

Figure S1 **Mathematical simulation of “electromigration” of AChRs during electric field-induced clustering.** Particle positions at the end of the simulation (filled circles), with initial positions (open circles) generated randomly. When DC electric field strength was set at 7.5 V/cm or less (A-D), similar to the level used in real experiments, final positions of particles showed no preferential localization. However, at very high field strength (2,000 V/cm or 20,000 V/cm), which are not physiological nor used experimentally, particles did become aggregated along the designated cathodal edge (E-F). From particle trajectories generated throughout the simulation period, Hurst exponents, Hurst exponent ratios, diffusion-coefficients and diffusion-coefficient ratios were calculated. Up to 7.5 V/cm field strength, none of these were significantly different from values at 0 V/cm, but when the field strength was set to 2,000 V/cm or 20,000 V/cm, the calculated values were greater than that obtained with 0 V/cm (G-J). For each electric field strength, data from 50 runs were pooled. Error bars indicate SEM. *p<0.01. The electric field direction is shown by the arrow in D.
(TIF)

Figure S2 **Simulation of the diffusion-trap model.** The cathodal edge on the right boundary of the simulation area was taken to be “absorbing” or “sticking”, mimicking the trap that immobilizes AChRs. After a 1,000-step run, most of the ten initially mobile AChRs (open circles) were immobilized at the cathodal edge (closed circles).
(TIF)

Text S1 Simulation of surface AChR movement.
(DOC)
