# Supplementary figures and images for: Mechanism of Acetylcholine Receptor Cluster Formation Induced by DC Electric Field
Source: PLoS One. 2011 Oct 25;6(10):e26805. doi: 10.1371/journal.pone.0026805 (PMC3201969; doi:10.1371/journal.pone.0026805)

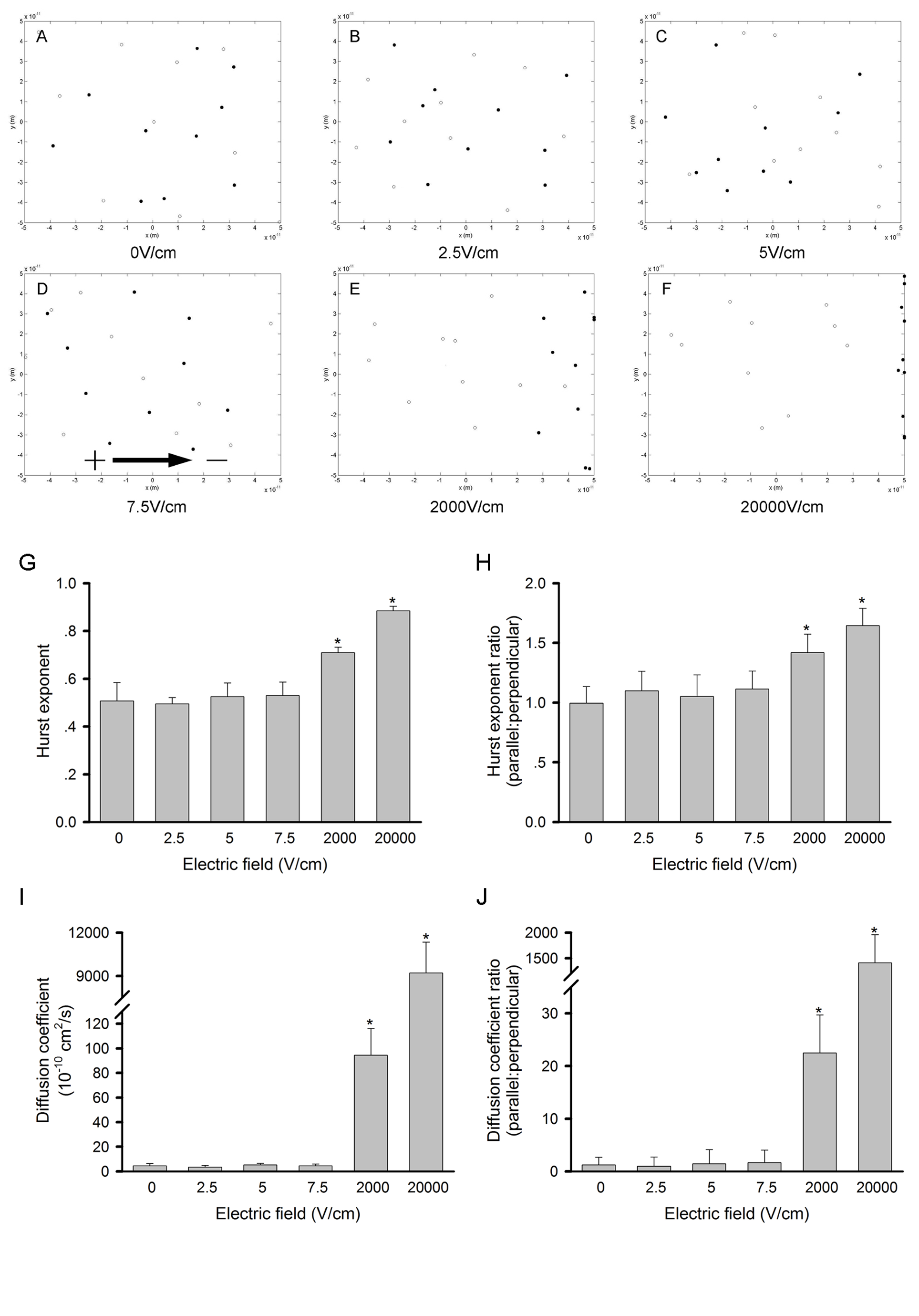

Supplement: Figure S1 — Mathematical simulation of “electromigration” of AChRs during electric field-induced clustering. Particle positions at the end of the simulation (filled circles), with initial positions (open circles) generated randomly. When DC electric field strength was set at 7.5 V/cm or less (A-D), similar to the level used in real experiments, final positions of particles showed no preferential localization. However, at very high field strength (2,000 V/cm or 20,000 V/cm), which are not physiological nor used experimentally, particles did become aggregated along the designated cathodal edge (E-F). From particle trajectories generated throughout the simulation period, Hurst exponents, Hurst exponent ratios, diffusion-coefficients and diffusion-coefficient ratios were calculated. Up to 7.5 V/cm field strength, none of these were significantly different from values at 0 V/cm, but when the field strength was set to 2,000 V/cm or 20,000 V/cm, the calculated values were greater than that obtained with 0 V/cm (G-J). For each electric field strength, data from 50 runs were pooled. Error bars indicate SEM. *p<0.01. The electric field direction is shown by the arrow in D. (TIF) [file pone.0026805.s001.tif]

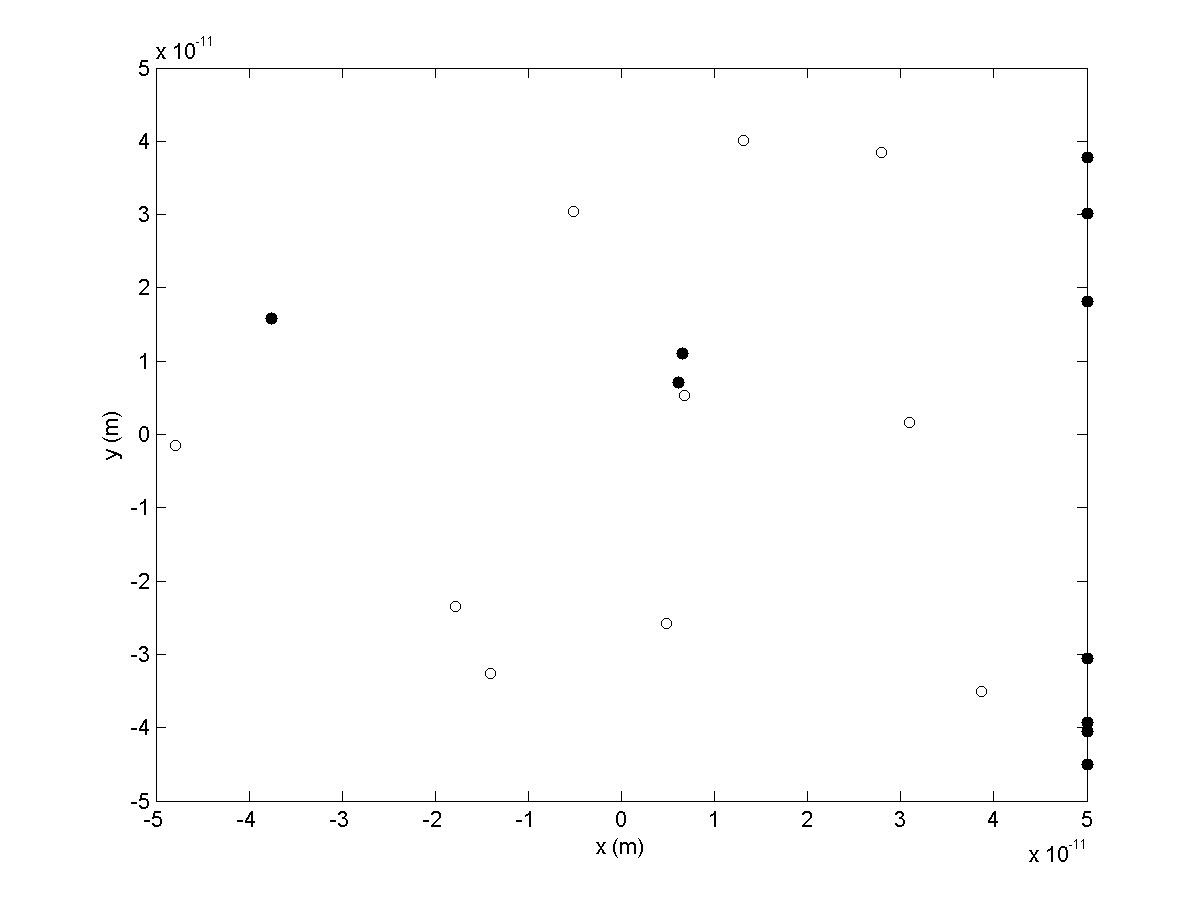

Supplement: Figure S2 — Simulation of the diffusion-trap model. The cathodal edge on the right boundary of the simulation area was taken to be “absorbing” or “sticking”, mimicking the trap that immobilizes AChRs. After a 1,000-step run, most of the ten initially mobile AChRs (open circles) were immobilized at the cathodal edge (closed circles). (TIF) [file pone.0026805.s002.tif]
