# Supplementary material for: Mechanism of Acetylcholine Receptor Cluster Formation Induced by DC Electric Field
Source: PLoS One. 2011 Oct 25;6(10):e26805. doi: 10.1371/journal.pone.0026805 (PMC3201969; doi:10.1371/journal.pone.0026805)
Supplement: Text S1 — Simulation of surface AChR movement. (DOC) [file pone.0026805.s003.doc]

Supplemental information

Simulation of surface AChR movement

To simulate the movement of QD-labeled AChRs on the muscle cell membrane, a collision model is set up by modification of Brownian motion based on Newton’s second law of motion and the law of conservation of momentum. Movement of lipid molecules on cell membrane is considered to be driven by thermal energy and its root mean square velocity can be estimated by:

where *k* is Boltzmann constant and equals to 1.381*10^-23^ J/K, *T* is absolute temperature and is set at 300K, and *m* is mass of membrane lipid molecule estimated to be 10^-24^ Kg [1]. Collisions between QD-AChRs and neighboring lipid molecules are taken to be elastic and complies with Langevin’s equation [2]. The equation of motion is described below.

$$M\frac{d^{2}s}{dt^{2}}=f\left(t\right)+F\left(t\right)$$

*f(t)* is the force on QD-AChR caused by the random collision by the lipid molecule and *F(t)* is the force from viscous resistance. Simulation according to this equation can be done by calculations based on the following steps.

**(1)** The position of QD-AChR at the nth collision is (*x_n_,y_n_*) and initial value is taken to be (*0,0*). The velocity of QD-AChR just before the nth collision is $V_{n}$ with the components along horizontal and vertical axis as $V_{nx}$and$V_{ny}$, which are both initialized as $V_{nx}=V_{ny}=0$. The velocity of the particle just after the nth collision is $V_{n}^{'}$ with the components along horizontal and vertical axis as $V_{nx}^{'}$and $V_{ny}^{'}$.

**(2)** QD-AChR is hit by a mobile lipid molecule with velocity$v$, which has a value of mean square velocity$\left|v\right|$and random directionwithin the range $[0,2\pi]$with respect to the horizontal axis. The velocity of lipid molecule after collision is $v'$. During the course of collision, the velocity, *v_c_*, of the center-of-mass of the system of particles consisting of QD-AChR and lipid molecule is defined as

$$v_{c}=\frac{mv+MV}{m+M}$$

where *M* is mass of QD-AChR estimated to be 5*10^-22^ Kg. Considering the force from colliding lipid molecule first:

$$M\frac{d^{2}s}{dt^{2}}=f\left(t\right)$$

According to the law of conservation of momentum, this can be described as:

$$m(v-v_{c})+M(V_{n}-v_{c})=m(v'-v_{c})+M(V_{n}^{'}-v_{c})=0$$

Meanwhile the law of conservation of energy should be applied,

$$\frac{1}{2}m(v-v_{c})^{2}+\frac{1}{2}M(V_{n}-v_{c})^{2}=\frac{1}{2}m(v'-v_{c})^{2}+\frac{1}{2}M(V_{n}^{'}-v_{c})^{2}$$

This gives

**(3)** During the time interval between two sequential hits, the motion of QD-AChR is affected by isotropic viscosity resistance calculated by Stokes law [3]. This component of the force on QD-AChR is as follows:

$$\begin{matrix} M\frac{d^{2}s}{dt^{2}}=F\left(t\right) \\ F(t)=-6\pi\eta\text{V}r \end{matrix}$$

The radius *r* is about 10^-8^ m (10 nm) and the viscosity of water *η* is estimated to be 0.001 pascal-second (pa-s). The velocity of QD-AChR at the end of the time interval just before the next collision is:

$$V_{(n+1)x}=V'_{nx}e^{-\frac{6\pi\eta r}{M}t}$$

$$V_{(n+1)y}=V'_{ny}e^{-\frac{6\pi\eta r}{M}t}$$

By integrating the velocity over the time interval, the position of BBQ at the end of each time interval is:

In the experiments carried out in this study, QD-AChRs were tracked at time interval of 0.1~0.5 s and within an area of about 10^-5^×10^-5^ m^2^. Thus, the simulations were performed with 1,000 steps per run, using a step time of 10^-11^ s and a domain of 10^-10^ ×10^-10^ m^2^, with these values being in proportion to the temporal and spatial scale used in the experiments. The boundaries of the simulation area are set to be elastic. This means that only the velocity component perpendicular to boundary will reverse its direction but the parallel component remains unaffected.

**Simulation of the electromigration model:**

For this model, DC electric field is applied along horizontal direction with positive infinity end as cathode. The combined effect of electrophoresis and electro-osmosis is described as a constant external force [3]:

$$F=6\pi r\varepsilon(\zeta_{AChR}-\zeta_{membrane})E$$

where ε is the Faraday constant 7.2×10^-10^ F/m and *ζ_membrane_* is the average cell membrane *ζ* (zeta) potential and is usually within a range -50 mV~-10 mV [4-7]. *ζ_AChR_* represents *ζ* potential of QD-AChR complex. The isoelectric point of BTX-AChR complex of about 5.00 gives a negative value of *ζ* potential in a culture medium with pH = 7.4 [6]. The *ζ* potential of QDs coated with streptavidin is approximately -25 mV [8]. So *ζ_AChR_* should be a negative value. When examining the maximum effect of DC electric field, *ζ_membrane_* and *ζ_AChR_* were set to be -50 mV and 0 mV respectively.

After the addition of external force from DC electric field, Langevin’s equation is modified to:

$$m\frac{d^{2}x}{dt^{2}}=f\left(t\right)+F\left(t\right)+6\pi\text{ }r\varepsilon(\zeta_{BBQ}-\zeta_{membrane})E$$

This leads to a modification of the parameters in step (3) as:

$$V_{(n+1)x}=V'_{nx}e^{-\frac{6\pi\eta r}{M}t}+\frac{6\pi\text{ }r\varepsilon(\zeta_{BBQ}-\zeta_{membrane})E}{M}t$$

$$V_{(n+1)y}=V'_{ny}e^{-\frac{6\pi\eta r}{M}t}$$

$$y_{n+1}=y_{n}+\frac{V'_{ny}M}{6\pi\eta r}(1-e^{-\frac{6\pi\eta r}{M}t})$$

(Note that the y-axis is devoid of the influence of the electric field.)

Results of the simulation are shown in Fig. S1. It can be noted that under the electric field (<10 V/cm) used in the present study, QD-AChRs are not preferentially sequestered toward the cathode at the end of the simulated tracking period (Fig. S1, A-D). The calculated Hurst exponents of 0.5 show the Brownian nature of particle movement under the electric field as observed experimentally (Fig. S1, G-H). As expected, the diffusion coefficients are independent of field strengths at these levels (Fig. S1, I-J). To determine if electrophoresis and electro-osmosis can theoretically influence AChR movement at high field strengths, the simulation was also conducted under the hypothetical field strength of 2,000 and 20,000 V/cm applied to the cells. As shown in Fig. S1 (E-F), cathodal AChR clustering is indeed achieved at these field strengths. This is accompanied by a significant increase in Hurst exponent towards 1.0 and also an increase in the diffusion coefficient with both biased toward the direction parallel to the field axis (Fig. S1, G-J). These simulation results support the conclusion that DC electric fields can only directly affect AChR aggregation by physicochemical action at very high strengths well beyond the physiological range. At physiological field strengths (<10 V/cm), it has no direct effect on receptor movement. In other words, the induction of AChR clustering by electric fields under physiological conditions does not involve the electromigration of receptors.

**Simulation of the diffusion-trapping model:**

For this simulation, the cathodal boundary is now considered to be “absorbing” or “sticking”, with others remaining elastic, to simulate the trap induced by the electric field. A 1,000-step run (with duration of 10^-8^ s) results in the appearance of cathodal AChR aggregates, consistent with our experimental results (Fig. S2). Thus, the diffusion-trap model offers a good explanation of the DC electric field-induced AChR clustering process according to this simulation.

#### References

#### 1. Aragones-Munoz A, Sandoval-Villalbazo A (2010) The relativistic Brownian motion: interdisciplinary applications. J Physics: Conference Series 229: 012075.

#### 2. Lemons DS, Gythiel A (1997) Paul Langevin's 1908 paper "On the Theory of Brownian Motion". Am J Phys 65: 1079-1081.

#### 3. McLaughlin S, Poo M-M (1981) The role of electro-osmosis in the electric-field-induced movement of charge macromolecules on the surfaces of cells. Biophys J 34: 85-93.

#### 4. Adak S, Chowdhury S, Bhattacharyya M (2008) Dynamic and electrokinetic behavior of erythrocyte membrane in diabetes mellitus and diabetic cardiovascular disease. Biochim Biophys Acta 1780: 108-115.

#### 5. Filek M, Zembala M, Szechynska-Hebda M (2002) The influence of phytohormones on zeta potential and electrokinetic charges of winter wheat cells. Z Naturforsch C 57: 696-704.

#### 6. Oswald RE, Freeman JA (1979) Characterization of the nicotinic acetylcholine receptor isolated from goldfish brain. J Biol Chem 254: 3419-3426.

#### 7. Zhang Y, Yang M, Portney NG, Cui D, Budak G et al. (2008) Zeta potential: a surface electrical characteristic to probe the interaction of nanoparticles with normal and cancer human breast epithelial cells. Biomed Microdevices 10: 321-328.

#### 8. Rife JC, Long JP, Wilkinson J, Whitman LJ (2009) Particle tracking single protein-functionalized quantum dot diffusion and binding at silica surfaces. Langmuir 25: 3509-3518.
